# Long-term impact of legume-inclusive diversification and nutrient management practices on phosphorus dynamics in alkaline *Fluvisol*

**DOI:** 10.1038/s41598-023-49616-x

**Published:** 2024-01-02

**Authors:** Asik Dutta, K. K. Hazra, C. P. Nath, N. Kumar, S. S. Singh, C. S. Praharaj

**Affiliations:** 1 Crop Production Division, Indian Institute of Pulses Research (ICAR), Kanpur, Uttar Pradesh 208024 India; 2 Rani Lakshmi Bai Central Agricultural University, Jhansi, Uttar Pradesh 284003 India; 3 Directorate of Groundnut Research, Junagadh, Gujarat 362001 India

**Keywords:** Agroecology, Environmental chemistry, Element cycles

## Abstract

An insight into the dynamics of soil phosphorus (P) pools with long-term cropping/management practices would help in designing efficient and sustainable management module(s). The study aimed to investigate the long-term impact of diversified rice-based rotations and variable nutrient management practices on the dynamic composition of P pools and their influence on systems’ base-crop productivity in an alkaline soil of Indo-Gangetic plain (*Fluvisol*). Treatments consisted of four rotations [rice–wheat (R–W), rice–wheat–mungbean (R–W–Mb), rice–wheat–rice–chickpea (R–W–R–C), rice–chickpea (R–C)] each with three nutrient treatments [control (CT), integrated nutrient management (INM), sole-chemical fertilizers (CF)]. Notably, R–C exhibited higher levels of bioavailable-P (soluble-P, Ca_2_-P, labile-Po), particularly in subsurface soil depth (0.2–0.4 m) compared to other rotations. Likewise, the inclusion of chickpea every alternate year (R–W–R–C) resulted in higher Ca_2_-P (40%), labile-Pi (15%), labile-Po (11%), and moderately labile Po (8%) compared to R–W rotation demonstrating an increased significance of chickpea in maintaining a favorable soil P regime in alkaline soil. Both R–C and R–W–R–C reduced the surface-to-subsurface depth ratio (SSBR) of soluble-P and Ca_2_-P while increasing the ratio for microbial biomass P. Even with a suboptimal fertilizer-P rate, INM significantly increased soluble-P (4–33%), labile-Po (13–17%), microbial biomass P (10–26%), moderately labile-Po (4–17%) compared to CF and exhibited higher SSBR values. Correlation analysis demonstrated the substantial influence of very-labile carbon, microbial and phosphatase activities on P availability. The treatment-induced changes in labile-P pools significantly influenced rice (base-crop) yields. In conclusion, chickpea-inclusive diversification and INM could be a sustainable approach to enhance P bioavailability and crop productivity in tropical rice soils.

## Introduction

Continuous rice–wheat (R–W) system is the predominant cropping practice in South Asia, currently facing multi-pronged sustainability challenges including nutrient mining, soil quality degradation, and groundwater depletion^1,2^. The continuous cereal-cereal rotation(s) without species diversification and imprudent fertilizer application has raised serious concerns regarding nutrient bioavailability and use efficiency^3^. Nitrogen (N) and phosphorus (P) are the two most important nutrients for crop growth associated in countless biochemical and physiological functions like energy conversion, translocation of photosynthates, root growth and so on^4^. Even, P-is referred as the “king-pin” of Indian agriculture and the indispensable part of the “energy currency” of plants^5^. However, P deficiency is a significant yield-limiting factor in tropical agro-regions, followed by N^5^. In particular, P deficiency is widespread in R–W systems, with P recovery efficiency often recorded below 30%^5^. Soil samples collected from 257 districts out of 500 districts (51%) across India, showed low available–P and the deficiency was dominated in R–W growing belts of Northern India^5^. Furthermore, the critical limit of P in rice and wheat range between 10.6–11.8 mg kg^−1^ and 3.3–7.9 mg kg^−1^ in the alluvial soil, respectively, and the soil’s inability to supply the required P level leads to sub-optimal yield^5^. Soil P bioavailability and its dynamic composition are contingent upon various soil chemical properties [e.g. pH, relative abundance of calcium (Ca), iron (Fe), and aluminum (Al) compounds], microbial activity, and moisture conditions^6^. In alkaline soils, the preeminent factor governing soil P availability is the presence of various Ca compounds, sometimes coated with Al/Fe oxides, on the surface of calcium carbonate (CaCO_3_)^7^. The speciation of P in the solid phase of the soil represents a critical means of quantifying the relative abundance of inorganic P (Pi) and organic P (Po), particularly in calcareous soils where Po constitutes the dominant pool, accounting for approximately 80% of the total P content^7^. Specifically in lowland rice ecologies, where flooding-induced P dissolution is a common phenomenon, soil-P fractionation would be a great approach for examining the inter-conversion among different Pi and Po fractions and their relationships with P availability^8^. Given the compelling evidence from previous studies, an insight into soil processes related to long-term soil/crop management practices in rice production systems is vital for developing crop/nutrient management option(s) to sustain soil fertility.

Species diversification could be a promising approach to restore soil fertility and mitigating the detrimental consequences associated with monoculture practices^9^. Grain legumes endowed with intrinsic characteristics like deep roots, biological N-fixation (BNF), higher root cation exchange capacity, the release of root exudates, and C rhizo-deposition, favors P cycling and increase P bioavailability^10,11^. Previous Research has demonstrated that legumes exhibit greater efficiency in mobilizing P compared to cereals and, consequently they can access non-labile P through mechanisms like rhizospheric acidification, symbiotic association with other arbuscular mycorrhizae, higher specific root length and P-transporter activity in the roots^12,13^. However, very few attempts have been taken till date to find out the relative dynamics of different soil P-fractions (both Pi and Po) in alkaline soils under legume based long run systems^14^. Consequently, there is a critical gap in understanding the long-term impacts of legume inclusion in agricultural systems on P bioavailability and dynamic composition of P pools, particularly in alkaline rice soils.

Sole chemical fertilizer-based nutrient management was found unsustainable in the long run and the reasons were: 1. increasing cost of chemical fertilizers leading to imbalanced application. 2. Disparity between domestic production and use. 3. Rise in soil pollution with concomitant ruination of soil health and groundwater quality. 4. Loss of soil biodiversity with rise of multi-nutrient deficiency and 5. Stagnation in soil productivity^15^. Integrated nutrient management with organic amendments can enhance soil biochemical processes, facilitating P mobilization, and their effectiveness depends on the quality and quantity of organic inputs, soil biochemical properties, and climatic factors. Organic matter plays a crucial role in improving P solubility and reducing P fixation, thereby enhancing P availability to plants^15^. Integrated nutrient management involving organic amendments, phosphate-solubilizing microorganisms, cover cropping, or crop species with higher P use efficiency has been suggested as a sustainable option for efficient P management^16–18^. Hence, it would be interesting to compare the effectiveness of *in-situ* recycling of cereal and legume residues on P cycling. Sequential extraction of P provides critical insights into P fluxes and the estimation of the fate of P inputs in soils^19^. Estimating lability-graded organic P pools is important to determine the soil’s P supply capacity depending on microbial processes. Therefore, an in-depth investigation of the impact of long-term species diversification in lowland flooded-rice systems and variable nutrient management practices on organic and inorganic P pools, as well as P-biogeochemistry, would provide valuable information for addressing both agronomic and environmental concerns^20^.

The present investigation was undertaken from a 14-year-long experiment aimed at unraveling the dynamic composition of soil P pools and their inter-relations as influenced by long-term rice-based systems with or without species (legume) diversification and variable nutrient management practices. Here, we hypothesized that (i) legume-inclusive rice-based rotation(s) would increase soil bioavailable P when compared to the conventional R–W rotation in the long run, (ii) implementing integrated nutrient management involving suboptimal fertilization rates (half of the recommended), crop residue recycling, and bio-fertilizer application would increase P bioavailability over sole chemical fertilization in alkaline rice soil, (iii) in the long run, integrated nutrient management would enrich P pools in surface soil depth compared to subsurface soil depths, resulting in higher values of surface-to-subsurface soil depth ratio (SSBR) of P pools, (iv) Treatments-induced changes in bioavailable P would have a significant positive correlation with the base-crop (rice) yield in long-run.

## Materials and methods

### Site characteristics

**Figure 1:**
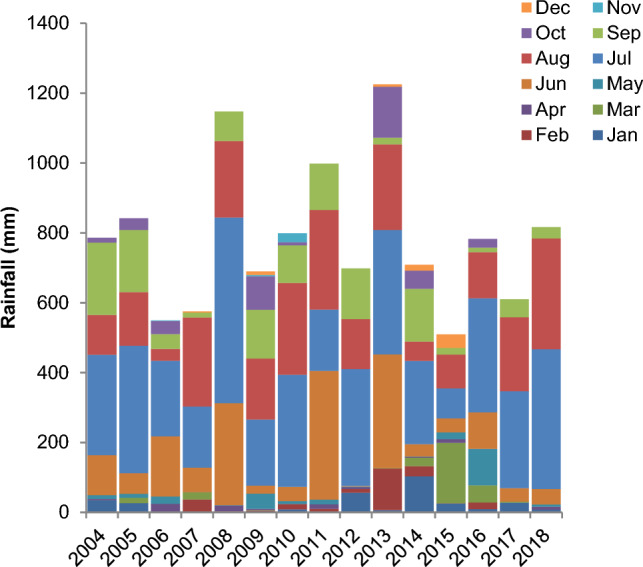
Month-wise distribution of annual rainfall (cm) of the experimental site (2004–2018).

The data was generated from a 14-year-old long-term experiment, maintained since June 2003 at the main research farm of ICAR-Indian Institute of Pulses Research, Kanpur, India. The climate of the study region is sub-tropical humid and characterized by hot summer and cool winter, with a mean annual rainfall of 722-mm. The rainfall mostly occurs between July–September with mean annual ambient temperature of 26 °C. The maximum and minimum temperature of Kanpur varies from 7 to 41.6 °C, respectively. The month-wise rainfall pattern during the experimental period is presented in Fig. 1. The experimental soil belongs to the order *Fluvisol* (World Reference Based soil classification^21^). The soil is sandy-loam in texture and moderately alkaline in reaction [pH 8.1 (1:2.5 soil-suspension ratio)]. At the initiation of the experiment (year 2003), the soil had low soil organic carbon (2.47 g kg^−1^)^22^ and available N (KMnO_4_-N: 100.4 mg kg^−1^)^23^, low available P (Olsen-P: 7 mg kg^−1^) and available potassium (K) (NH_4_OAc-K: 79.6 mg kg^−1^)^24^.

### Treatments and experimental design

**Figure 2:**
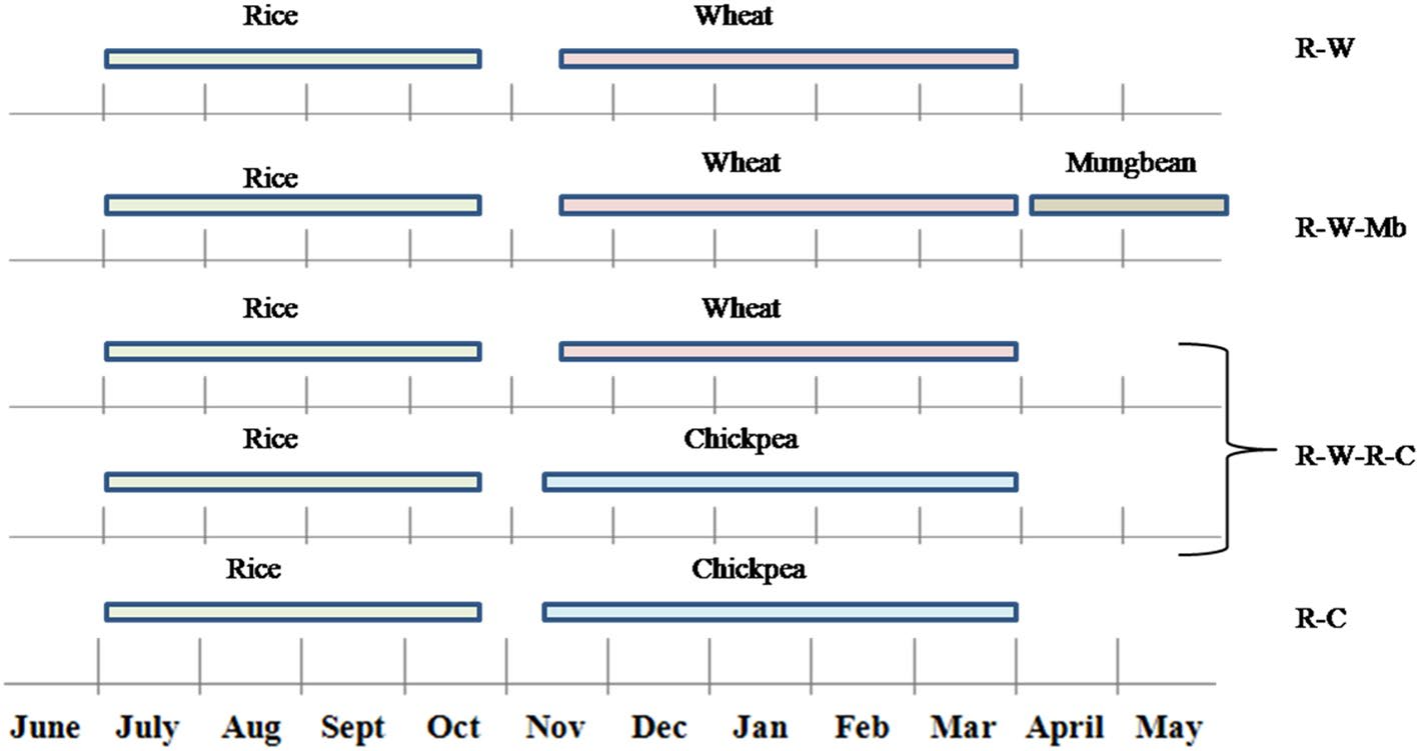
Temporal distribution of component crops under different crop rotations in the long-term field trial (Kanpur, India).

The treatments comprised four crop rotations i.e., [(i) rice–wheat (R–W), (ii) rice–wheat–mungbean (R–W–Mb), (iii) rice–wheat–rice–chickpea (R–W–R–C) (2 years rotation), and (iv) rice–chickpea (R–C)] each with three levels of nutrient management treatments [(i) without any chemical and organic fertilization or control (CT), (ii) recommended inorganic fertilizers comprising recommended rate of N, P, K, sulphur (S), zinc (Zn), and boron (B) (RDF), and (iii) integrated nutrient management (INM: half dose of the recommended/prescribed fertilizer rate of N, P, K+ crop residues recycling of all crops + farmyard manure (FYM) at 5 t ha^−1^ + bio-fertilizers)]. The temporal distribution of component crops in different rotations is presented in Fig. 2. The crop rotation and nutrient management treatments were randomly allocated in the main plot and subplots in split-plot design (SPD), and each treatment was replicated thrice. There were total of thirty-six plots, and each plot dimension was 11 m × 7 m. The detailed treatment description (fertilizer rate, fertilizer application timing, crop cultivars, crop sowing time, crop-wise irrigation frequency) and crop management is specified in Table 1. Each year ~ 15 days prior to sowing of the rainy season crops, well decomposed farmyard manure was incorporated uniformly by plough tillage in INM treatments. Bio-fertilizers (*Azotobacter* for rice and wheat, *Rhizobium* for chickpea and mungbean, and phosphate solubilizing bacteria *Bacillus polymyxa* for all crops) were applied (> 10^7^ bacteria g^−1^ culture) through seed treatment. Fertilizers urea (46% N), di-ammonium phosphate (18% N and 46% P_2_O_5_), muriate of potash (60% K_2_O), gypsum (23.5% S), zinc sulphate (21% Zn) and borax (11% B) were used as sources of N, P, K, S, Zn and B, respectively. The fertilizer-N applied through di-ammonium phosphate was calculated for each treatment and adjusted with urea N.

**Table 1:** General crop management practices adopted in different component crops in the long-term experiment.

Crop	Sowing month	Cultivar	Spacing (cm)	Seed rate (kg ha^−1^)	No of irrigation^$^	Nutrient management			Time of fertilizer application
						Control	INM	RDF	
Rice	July (1)#	Pant Dhan12	20 × 20	40	10	Nil	N–P_2_O_5_–K_2_O: 60–30–20 + Full crop residue of previous crop + FYM 5 Mg ha^−1^ + biofertilizers 20 g kg^−1^ seed	120–60–40:20:5:1.1 (N:P_2_O_5_:K_2_O:S:Zn:B kg ha^−1^)	1/3 N + P + K at basal;1/3 N at 25 DAT; 1/3 N at 45 DAT
Wheat	November (3)	PBW 343 /HD 2967	22.5 cont	100	5	Nil	N–P_2_O_5_–K_2_O: 60–30–20 + Full crop residue of previous crop + biofertilizers 20 g kg^−1^ seed	120–60–40 (N:P_2_O_5_:K_2_O kg ha^−1^)	1/3 N + P + K at basal; 1/3 N at 21 DAS; 1/3 N at 45 DAS
Mungbean	April (1)	Samrat (PDM 139)	30 × 10	12	4	Nil	N–P_2_O_5_–K_2_O: 10–30–20 + Full crop residue of previous crop + biofertilizers 20 g kg^−1^ seed	20–60–40 (N:P_2_O_5_:K_2_O kg ha^−1^)	N + P + K at basal
Chickpea	November (2/3)	KWR 108/JG 16	30 × 10	70	2	Nil	N–P_2_O_5_–K_2_O: 10–30–20 + Full crop residue of previous crop + biofertilizers 20 g kg^−1^ seed	20–60–40 (N:P_2_O_5_:K_2_O kg ha^−1^)	N + P + K at basal

*INM*, integrated nutrient management; *RDF*, recommended dose of chemical fertilizers; *DAT*, days after transplanting; *DAS*, days after sowing. # value in the parenthesis represents the week of the month; $ need-based irrigation frequency was calculated based on last four years median data (2014–2018).

### Crop management practices

For rice crop, the field was ploughed twice followed by harrowing and planking. Wet tillage (puddling) was performed 1 day before transplanting. Twenty-five days-old rice seedlings were planted in the main field. Three seedlings per hill were planted. The flooding method of irrigation was employed for the rice crop, with each irrigation event providing a water depth of 8–10 cm. Insecticide chlorpyrifos was applied with irrigation water to control termites. In preparation for the winter crops (wheat and chickpea), the plots were ploughed, harrowed, and planked. In both rainy and winter crops, hand weeding was done at 25 and 45 days after sowing to maintain weed-free conditions. In the summer season, immediately after wheat harvest, mungbean crop was established in R–W–Mb rotation. Before mungbean sowing, the plots were prepared with harrowing and planking. Necessary plant protection measures were taken in all crops to grow disease and insect pest-free crops. Wheat, chickpea, and mungbean were irrigated using the check basin method, with each irrigation event providing a depth of 6 cm of water.

### Soil sampling and processing

Soil was collected after 14 years of long-term cropping (2017) i.e., at the harvest of the base crop (rice). From each treatment plot, six representative field-moist soil samples were taken from 0–0.2 m and 0.2–0.4 m depths with a posthole auger. The soils of different sampling points were collected separately and mixed thoroughly to get the representative composite sample for each treatment (*n* = 36). All the collected samples were air-dried, ground, sieved (0.2-mm sieve), processed to prepare final samples and each sample was divided into two sample sets. One subset was kept in the refrigerator at 4 °C for analysis of microbiological parameters and a second subset was stored in normal room temperature (~ 25 °C) for analyzing different chemical parameters.

### Analysis soil P pools

#### Determination of soil inorganic P (Pi) fractions

The study employed an inorganic P (Pi) fractionation scheme based on differential solubility in various extracts. The Pi fractionation scheme primarily followed the procedure outlined by Kuo^25^, with certain modifications made to extract the Ca-bound P fractions^26^. The Pi fractions were categorized into seven pools viz. soluble-P (Sol-P), di-calcium P (Ca_2_-P), octa-calcium P (Ca_8_-P), aluminum P (Al-P), iron P (Fe-P), occluded P (Occl-P), deca-calcium P (Ca_10_-P). For this, 0.5 g soil sample was taken in a 50 ml centrifuge tube. The first fraction i.e*.*, soluble and loosely bound Pi (Sol-P), was extracted by shaking the soil sample in 1 M ammonium chloride (NH_4_Cl) solution (25 ml) for 30 min. Di-calcium bound P (Ca_2_-P) encompasses water soluble-P, citrate soluble-P, partially surface adsorbed P and considered available for plants. The di-calcium bound P fraction was extracted using 25 ml 0.25 M sodium bicarbonate (NaHCO_3_) (pH 7.5) and shaken for 1 h. The third fraction viz*.* octa-calcium bound Pi (Ca_8_-P), is classified as sparingly soluble P. Ca_8_-P was extracted by shaking the soil with 25 ml ammonium acetate (C_2_H_7_NO_2_) (pH 4.2) for 1 h and then soil sample was washed with 25 ml of 95% methanol for 15 min. The Al-bound P fraction (Al-P) and Fe-bound fraction (Fe-P) are usually non-available P but under extremely P depleted conditions, plants can utilize these P pools. Al-P was separated using 0.5 M ammonium fluoride (NH_4_F) (pH 8.2) with 1 h of shaking, while, Fe-P was extracted by 0.1 M sodium hydroxide (NaOH) with 17 h of shaking. After completion of both steps, soil samples were washed with 25 ml saturated NaCl solution. Occluded P (Occ-P) found within the matrices of retaining aggregates and minerals, was extracted using CDB [sodium citrate (Na_3_C_6_H_5_O_7_·2H_2_O)-sodium dithionate (Na_2_S_2_O_4_)-sodium bicarbonate] extraction. For this fraction, 20 ml of 0.3 M Na_3_C_6_H_5_O_7_ 2H_2_O and 5 ml of 1 M NaHCO_3_ were added to the soil and shaken for 15 min with subsequent heating in a water bath for 15 min at 85 °C. After that, 0.5 g Na_2_S_2_O_4_ was added and stirred rapidly to extract occluded P. As before, the samples were washed with 25 ml NaCl solution and after adjusting the final volume, the samples were kept in a well-aerated place overnight. Lastly, Ca_10_-P that represents a group of phosphate with chemical structure similar to hydroxyl apatite [Ca_10_(PO_4_)_6_·(OH)_2_], was extracted using 25 ml 0.25 M sulphuric acid (H_2_SO_4_) for 1 h followed by washing with saturated NaCl for 15 min. In each step, after extraction and washing, the supernatant was decanted into a 50 ml volumetric flask, and volume adjustment was carried out using deionized water. In each step, 10 ml of supernatant was taken in a 25 ml volumetric flask and P concentration was determined using phospho-molybdate method^27^.

#### Determination of organic P fractions/pools

The study employed an organic P (Po) fractionation scheme initially developed by Bowman and Cole^28^, with subsequent modifications introduced by Ivanoff^29^. The Po fraction could generally be differentiated into labile Po, moderately labile Po, and non-labile Po. For extraction of labile-Po, duplicate samples of 0.5 g were taken, and the P concentration in the extracts was determined colorimetrically using the phospho-molybdate method. In one sample, 0.5 M NaHCO_3_ (pH 8.5) was added and shaken for 16 h. The final volume of the aliquot was maintained at 50 ml after completing preceding steps such as shaking, centrifugation, and filtration. The labile Pi was determined using the method proposed by Murphy and Riley^27^, and the total labile Pi was measured after an aliquot (5 ml) was digested with 2.5 M H_2_SO_4_ and potassium persulfate (K_2_S_2_O_8_)^30^. The difference between the total labile P following persulfate oxidation and labile Pi provided an estimate of labile Po. Microbial biomass phosphorus (MBP) in the soil was determined using the chloroform (CHCl_3_) fumigation method. Duplicate 0.5 g samples were treated with 2 ml ethanol-free chloroform (CHCl_3_) and incubated under a fume hood for 24 h. After incubation, soil samples were extracted using 0.5 M NaHCO_3_, as mentioned previously. The difference between the amounts of total labile P in the CHCl_3_-treated and untreated soil samples provided an estimate of biomass P originated from lysed microbial cells^31^.

The determination of moderate Po involved a two-step process. First, extraction of moderately labile Po was carried out by shaking the residual soil sample in 1 M hydrochloric acid (HCl) for 3 h and then subtracting total P from moderately labile Pi, as discussed above. Second, the samples were washed with deionized water, and the supernatant, after centrifugation, was discarded. After that, 25 ml 0.5 M NaOH solution was added to the residue and after 3 h of shaking and centrifugation, the supernatant was collected. This supernatant contains both humic acid-Po and fulvic acid-Po. To separate these two fractions, one portion of the supernatant was acidified to pH 1–1.5 using concentrated HCl, where only fulvic acids remained in solution and humic acid precipitated. Total P was then analyzed using the persulfate oxidation method in both samples *i.e.* acidified (measures of fulvic acid Po) and non-acidified NaOH extract. The total P in the acidified sample provided the value for fulvic acid Po, whereas the subtraction of the total P measured in the NaOH extract and the fulvic acid Po provided the measure of humic acid Po.

### Soil enzyme analyses

Acid and alkaline phosphatases were determined following the methodology outlined by Tabatabai and Bremner^32^, using 16 mM para (*p*)-nitrophenyl phosphate as substrate and reported as µg p-nitrophenol produced g^−1^ dry soil hour^−1^. Total organic carbon (TOC) was analyzed using a TOC analyzer (Multi N/C 2100, Analytikjena, Germany). To estimate TOC, the soil was first treated with HCl to eliminate soil inorganic carbon (calcium carbonates), and then, TOC was estimated following the dry combustion method^33^. The labile carbon pool was analyzed using modified Walkley and Black method^34^. The chloroform-fumigation extraction method was used for the estimation of microbial biomass carbon (MBC)^35^. Briefly, 20 g (dry weight equivalent) soil was fumigated with ethanol-free chloroform for 48 h. Both fumigated and non-fumigated soils were extracted with 50 ml of 0.5 M K_2_SO_4_, followed by shaking on an end-to-end shaker for 30 min. The organic carbon content of the extract was determined through oxidation with potassium dichromate^36^. The difference in the carbon content of the fumigated and non-fumigated extracts was multiplied by a factor of 0.33 to calculate MBC and was expressed as µg g^−1^ of dry soil^37^.

### Estimation of crop yield (base crop)

A net plot area of 37.8 m^2^ was designated for separate harvesting of grain and straw/stover yields. The harvested produce was subjected to sun drying, and the moisture content was recorded. The rice grain yield was subsequently adjusted to 14% moisture content and reported as tons per hectare (t/ha).

### Statistical analysis

The data were analyzed using the analysis of variance technique for a split-plot design, with crop rotation as main plot factor (with 4 levels) and nutrient management practices as sub-plot factor (with 3 levels), using the online statistical program OPSTAT^38^. Significance of treatment effects was assessed through the F-test. To compare treatment mean values, the least significance difference (LSD) value was utilized at a probability level of *p* ≤ 0.05. Principal component analysis (PCA) employed to explore the associations among different P pools in relation to the treatments, which encompassed crop rotation and nutrient management. PCA was performed using the PAST software (version 3.14). Additionally, cluster-cum-heatmap analysis was carried out using the online platform ClustVis 2.0^39^. The Pearson’s correlation coefficient (*r*) and regression models, both bi-variate and multivariate, were determined using Microsoft ExcelTM 2007 with the Data Analysis Toolpack.

## Results

### Inorganic P pools

Among the different Pi fractions, Ca_8_-P was the predominant fraction in both 0–0.2 m (surface soil) and 0.2–0.4 m (sub-surface soil) depths, respectively (Table 2). Notably, Ca_2_-P constituted 9% and 7% of total Ca-P in the 0–0.2 m and 0.2–0.4 m depth, respectively. Crop rotation effect was particularly pronounced on Ca_2_-P and Ca_10_-P in the surface soil. In the surface soil depth, the treatment trend for Ca_2_-P was as follows: R–C≥R–W > R–W–R–C ≥ R–W–Mb (*p* ≤ 0.05), while in the subsurface soil depth, it was R–C ≥ R–W–R–C ≥ R–W ≥ R–W–Mb (*p* ≤ 0.05). Compared to the conventional R–W system, all legume-inclusive rotations depleted Ca_8_-P fraction, maximum with R–W–Mb (17%). R–C rotation had a higher Ca_10_-P (25–29%) in the surface soil depth when compared to other rotations. Furthermore, R–W and R–C rotations led to a reduction in occluded-P in the subsurface soil depth (5.9–6.0 mg kg^−1^) compared to R–W–Mb and R–W–R–C rotations (7.1–7.9 mg kg^−1^) (*p* ≤ 0.05). Crop rotation treatments did not significantly alter Al-P and Fe-P fractions (Table 2).

**Table 2:** Inorganic fractions of soil phosphorus at surface (0–0.2 m) and subsurface (0.2–0.4 m) depths as influenced by long-term crop rotation and nutrient management practices.

Soil depth	Treatment	Soluble-P	Ca_2_-P	Ca_8_-P	Al-P	Fe–P	Occluded-P	Ca_10_-P
0–0.2 m	Crop rotation (CR)	(mg kg^−1^)						
	R–W	8.1 ± 0.2	8.4 ± 0.2	51.3 ± 1.7	13.5 ± 0.3	11.3 ± 0.2	14.1 ± 0.2	22.0 ± 0.2
	R–W–Mb	6.8 ± 0.3	7.2 ± 0.1	55.3 ± 2.0	11.5 ± 0.6	11.1 ± 0.1	15.1 ± 0.1	22.7 ± 0.1
	R–W–R–C	7.6 ± 0.1	7.6 ± 0.0	54.2 ± 0.9	13.1 ± 0.5	11.4 ± 0.6	15.8 ± 0.3	21.9 ± 0.4
	R–C	7.7 ± 0.5	8.6 ± 0.1	58.2 ± 3.2	12.9 ± 0.2	11.3 ± 0.3	15.5 ± 0.8	24.9 ± 0.8
	LSD (*p* = 0.05)	*ns*	0.73	*ns*	*ns*	*ns*	*ns*	2.26
	Nutrient management (NM)							
	CT	5.2 ± 0.1	5.4 ± 0.0	52.2 ± 1.9	12.3 ± 0.1	9.8 ± 0.1	15.8 ± 0.1	23.2 ± 0.3
	INM	8.9 ± 0.2	9.0 ± 0.0	55.0 ± 2.0	12.9 ± 0.1	11.5 ± 0.1	13.9 ± 0.1	22.8 ± 0.1
	CF	8.6 ± 0.1	9.5 ± 0.0	57.0 ± 0.1	13.1 ± 0.2	12.4 ± 0.1	15.7 ± 0.0	22.7 ± 0.1
	LSD (*p* = 0.05)	0.26	0.42	1.79	*ns*	0.58	0.86	1.82
	Interaction (*p* value)	< 0.001	< 0.001	< 0.001	0.019	0.009	0.033	< 0.001
	CR × NM							
0.2–0.4 m	Crop rotation (CR)							
	R–W	4.6 ± 0.1	4.3 ± 0.1	55.2 ± 0.2	9.9 ± 0.1	8.3 ± 0.1	5.9 ± 0.1	21.1 ± 0.2
	R–W–Mb	4.3 ± 0.1	4.2 ± 0.1	45.5 ± 0.4	10.6 ± 0.2	9.1 ± 0.4	7.9 ± 0.2	19.4 ± 0.1
	R–W–R–C	5.0 ± 0.0	6.0 ± 0.1	50.6 ± 1.9	11.2 ± 0.3	9.2 ± 0.6	7.1 ± 0.0	19.6 ± 0.2
	R–C	5.3 ± 0.1	5.8 ± 0.1	50.9 ± 0.2	11.3 ± 0.3	9.9 ± 0.3	6.0 ± 0.1	21.8 ± 1.3
	LSD (*p* = 0.05)	0.58	0.54	4.2	*ns*	*ns*	0.58	*ns*
	Nutrient management (NM)							
	CT	3.9 ± 0.1	3.8 ± 0.1	50.9 ± 0.4	10.2 ± 0.1	8.0 ± 0.2	6.1 ± 0.1	20.7 ± 0.7
	INM	6.1 ± 0.2	5.8 ± 0.1	52.6 ± 0.7	10.9 ± 0.2	9.4 ± 0.2	7.7 ± 02	21.4 ± 0.2
	CF	4.6 ± 0.1	5.7 ± 0.1	48.1 ± 0.1	11.1 ± 0.1	9.9 ± 0.0	6.3 ± 0.1	19.2 ± 0.1
	LSD (*p* = 0.05)	0.53	0.26	2.2	0.69	1.15	0.29	*ns*
	Interaction (*p* value)	< 0.001	< 0.001	*ns*	*ns*	*ns*	< 0.001	*ns*
	CR × NM							

*R–W*, rice–wheat; *R–W–Mb*, rice–wheat, mungbean; *R–W–R–C*, rice–wheat-rice-chickpea; *R–C*, rice-chickpea; *CT*, control; *INM*, integrated nutrient management; *CF*, sole chemical fertilizer treatment. Values represent mean ± standard error of mean; *LSD*, least significant difference; *ns,* non-significant.

The INM resulted in 4% and 33% increase in soluble-P compared to CF in the surface and subsurface soil depths, respectively (Table 2). Notably, INM led to a positive change in Ca_2_-P in surface soil depth when compared to CF. In the subsurface soil depth, INM plots exhibited higher levels of Ca_8_-P (9%) and occluded-P (22%) than CF. In contrast, CF treatment had a higher content of Ca_8_-P (4%), Fe-P (8%), and occluded-P (13%) than INM in the surface soil depth. The surface soil of CT plots exhibited significant depletions in sol-P (65%), Ca_2_-P (76%), Ca_8_-P (9%), and Fe-P (26%) in comparison to CF plots. Similarly, in the subsurface soil depth, notable depletions of sol-P, Ca_2_-P, and Al-P were observed in CT plots. However, CT treatment maintained a higher Ca_10_-P in the surface soil depth compared to INM and CF treatments (*p* ≤ 0.05). The interaction effect between crop rotation and nutrient management was significantly influenced all the inorganic P fractions of surface soil depths (*p* ≤ 0.05) (Table 2; Supplementary Fig. 1).

### Lability-graded inorganic and organic P pools

In the surface soil depth, the impact of crop rotation on labile-Pi was comparable, whereas the chickpea-inclusive rice-based rotations (R–C, R–W–R–C) increased labile-Pi by 12–15% in the subsurface soil depth compared to the R–W rotation (Table 3). Additionally, in the surface soil depth, R–C and R–W–Mb rotations exhibited a higher labile-Po pool than the R–W and R–W–R–C rotations (*p* ≤ 0.05). In the subsurface soil depth, both R–C and R–W–R–C rotations increased labile-Po by 11–13% compared to the R–W rotation, while R–W–Mb reduced labile-Po by 12% (*p* ≤ 0.05). Crop rotation treatments did not show significant differences in MBP in the surface soil depth (*p* > 0.05). However, in the subsurface soil depth, the R–W–Mb had a higher MBP (11%) compared to the R–W, whereas R–C and R–W–R–C decreased MBP. Only R–C rotation significantly increased moderately labile-Pi (14%) over the R–W rotation in subsurface soil depth. The order of moderately labile-Po followed the treatment sequence: R–W–Mb > R–W > R–W–R–C > R–C (*p* ≤ 0.05). Chickpea-inclusive rotations increased moderately labile-Po (8–11%) compared to R–W in the subsurface soil depth. The content of humic acid-Po was highest in the R–W–R–C rotation in the surface soil depth, while it was lowest in R–W–Mb. Likewise, a higher concentration of fulvic acid-Po was observed in chickpea-inclusive rotations compared to R–W and R–W–Mb rotations in the subsurface soil depth (Table 3).

**Table 3:** Lability-graded soil organic and inorganic P pools and microbial biomass P (MBP) at surface (0–0.2 m) and subsurface (0.2–0.4 m) depths as influenced by long-term crop rotation and nutrient management practices.

Soil depth	Treatment	Labile Pi	Labile Po	Microbial biomass P	Moderately labile Pi	Moderately labile Po	Humic acid Po	Fulvic acid Po
0–0.2 m	Crop rotation (CR)	(mg kg^−1^)						
	R–W	20.5 ± 0.3	54.5 ± 1.4	25.0 ± 0.6	55.7 ± 0.8	118.3 ± 1.4	33.6 ± 0.8	43.9 ± 1.5
	R–W–Mb	20.8 ± 0.9	59.5 ± 0.7	23.4 ± 0.1	54.4 ± 0.1	122.7 ± 1.8	29.0 ± 0.5	46.2 ± 0.4
	R–W–R–C	21.2 ± 0.9	54.2 ± 0.6	27.4 ± 0.3	53.4 ± 0.7	111.0 ± 0.1	38.6 ± 1.5	41.3 ± 1.2
	R–C	21.1 ± 0.1	61.1 ± 0.4	25.9 ± 1.9	53.7 ± 0.3	101.5 ± 0.1	34.2 ± 0.5	44.5 ± 0.6
	LSD (*p* = 0.05)	*ns*	2.20	*ns*	*ns*	4.41	4.99	*ns*
	Nutrient management (NM)							
	CT	14.7 ± 1.0	47.3 ± 0.7	10.6 ± 0.1	45.0 ± 0.5	102.5 ± 0.7	32.8 ± 0.5	39.1 ± 0.2
	INM	24.4 ± 0.9	67.1 ± 0.8	34.3 ± 1.4	58.1 ± 0.2	120.9 ± 0.3	36.1 ± 0.7	48.8 ± 0.6
	CF	23.7 ± 0.0	57.4 ± 0.0	31.3 ± 0.0	59.8 ± 0.0	116.7 ± 0.0	32.6 ± 0.0	44.1 ± 0.0
	LSD (*p* = 0.05)	2.12	2.4	2.5	1.50	2.7	1.42	1.42
	Interaction (*p* value)	*ns*	0.007	< 0.001	< 0.001	< 0.001	< 0.001	0.001
	CR × NM							
0.2–0.4 m	Crop rotation (CR)							
	R–W	13.0 ± 0.1	48.9 ± 0.7	19.9 ± 0.2	36.7 ± 0.5	81.2 ± 1.4	35.8 ± 0.4	33.2 ± 0.6
	R–W–Mb	14.0 ± 0.5	43.1 ± 1.3	21.1 ± 0.4	39.4 ± 0.1	83.6 ± 1.6	37.4 ± 0.2	33.0 ± 0.5
	R–W–R–C	14.9 ± 0.3	54.2 ± 1.7	18.0 ± 0.5	39.6 ± 0.2	87.8 ± 0.8	30.4 ± 0.0	37.4 ± 0.1
	R–C	14.6 ± 0.0	55.4 ± 0.2	14.4 ± 0.9	41.8 ± 1.0	90.3 ± 0.3	38.1 ± 0.2	35.8 ± 0.1
	LSD (*p* = 0.05)	1.17	5.0	1.23	3.01	5.84	1.42	2.17
	Nutrient management (NM)							
	CT	11.4 ± 0.4	45.1 ± 0.0	7.5 ± 1.0	34.4 ± 0.3	75.2 ± 1.3	35.1 ± 0.8	30.3 ± 0.5
	INM	16.6 ± 0.1	56.4 ± 1.1	26.5 ± 0.0	43.1 ± 1.1	98.1 ± 0.6	36.5 ± 0.0	40.0 ± 0.3
	CF	14.5 ± 0.0	49.8 ± 0.0	21.0 ± 0.0	40.7 ± 0.0	83.9 ± 0.0	34.7 ± 0.0	34.4 ± 0.0
	LSD (*p* = 0.05)	0.85	1.71	2.5	2.2	3.14	*ns*	1.52
	Interaction (*p* value)	0.009	< 0.001	0.023	< 0.001	< 0.001	< 0.001	0.004
	CR × NM							

Values represent mean ± standard error of mean; *LSD*, least significant difference; *ns,* non-significant.

The INM treatment increased labile-Po (17%), MBP (9%), moderately labile-Po (4%), humic acid-Po (11%), and fulvic acid-Po (11%) in the surface soil depth compared to the CF treatment (*p* ≤ 0.05) (Table 3). Similarly, significant incremental changes in labile-Pi (15%), labile-Po (13%), MBP (26%), moderately labile-Po (17%), and fulvic acid Po (16%) were observed in the subsurface soil depth in INM plots over the CF plots. In control plots, a marked decline was observed in all P forms in both surface and subsurface soil depths, with the most significant reductions noted for MBP (64–66%), labile-Pi (21–40%), and moderately labile-Pi (16–25%) (Table 3). The interaction between crop rotation and nutrient management was significant for most of the organic P fractions (*p* ≤ 0.05) (Table 3; Supplementary Fig. 1; Supplementary Fig. 2).

### Surface–subsurface allocation of P pools

**Figure 3:**
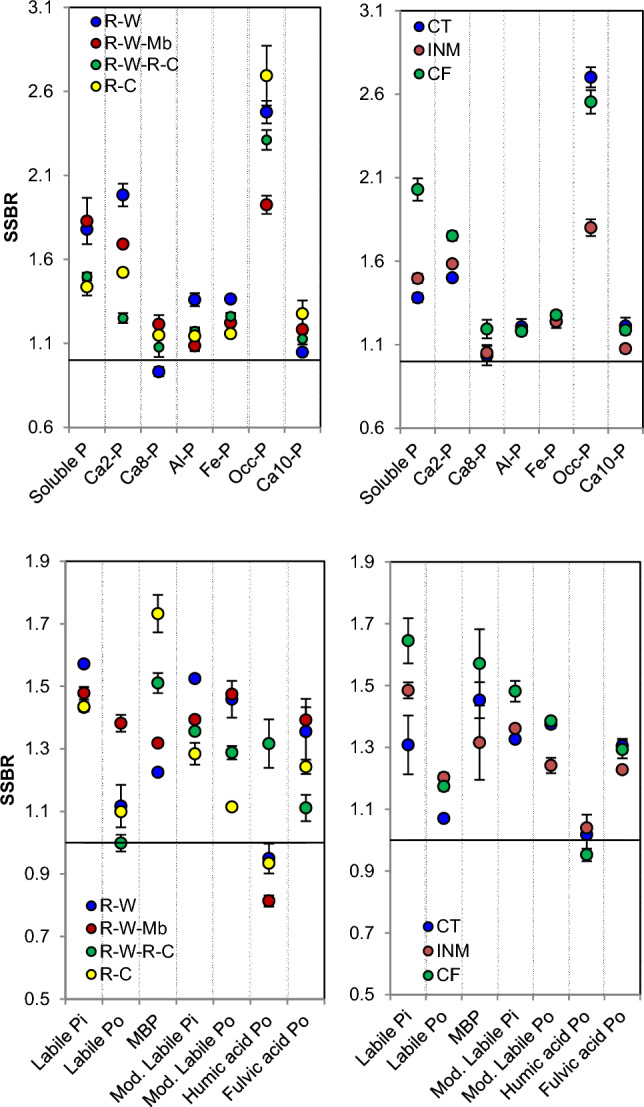
Surface soil and sub-surface soil depth ratio (SSBR) of different inorganic (**a**,**b**) and organic P (**c**,**d**) pools as influenced by long-term crop rotation and nutrient management treatments. Error bar represents standard error of means. *R–W*, rice–wheat; *R–W–Mb*, rice–wheat, mungbean; *R–W–R–C*, rice–wheat–rice–chickpea; *R–C*, rice-chickpea; *CT*, control; *INM*, integrated nutrient management; *CF*, sole chemical fertilizer treatment. *MBP*, microbial biomass phosphorus; *Pi*, inorganic phosphorus; *Po*, organic phosphorus.

The treatment effect was prominent on surface to subsurface soil depth ratio (SSBR) for various P pools (Fig. 3). Different rice-based rotations induced notable alterations in SSBR of several P pools, including sol-P (1.44–1.83), Ca_2_-P (1.25–1.98), occluded-P (1.93–2.69), labile-Po (1–1.38), moderately labile-Po (1.11–1.47), humic acid-Po (0.81–1.32), and fulvic acid-Po (1.11–1.39). Specifically, R–W and R–C rotations led to a decrease in SSBR for sol-P, Ca_2_-P, and moderately labile-Po, while increasing SSBR for MBP (*p* ≤ 0.05). Notably, R–C rotation significantly increased the SSBR of humic acid-Po in comparison to other rotations but decreased the SSBR of fulvic acid-Po. Among the rotations, R–W–Mb had the lowest SSBR (1.93) for occluded-P while SSBR for labile-Po (1.38) was the lowest. Furthermore, the CF treatment resulted in a higher SSBR for most of P pools compared to the INM, with notable increases observed in sol-P (+ 36%), labile-Pi (+ 11%), moderately labile-Po (12%). Among the various P pools, the mean SSBR was highest for occluded-P (2.35) (Fig. 3).

### Correlations and multivariate analysis

Strong positive correlations were observed among labile-Pi, labile-Po, and MBP in the surface soil depth (Table 4). In the surface soil depth, Fe–P pools exhibited higher positive correlations with inorganic P pools except for Ca_10_-P. Acid and alkaline phosphatases showed positive correlations with labile-Po but not with labile-Pi. MBC was positively correlated with MBP, labile-Pi and labile-Po in the surface soil depth. Furthermore, a positive correlation was evident between total organic carbon (TOC) and MBP (Table 4).

**Table 4:** Pearson correlation matrix of different soil P forms in surface (0–0.2 m) and subsurface (0.2–0.4 m) soil depths.

Surface depth (0–0.2 m)	1	2	3	4	5	6	7	8	9	10	11	12	13	14
1. Labile-Pi														
2. Labile-Po	0.83***													
3. Microbial biomass P	0.86***	0.62*												
4. Moderately labile-Pi	0.81**	0.61*	0.86***											
5. Moderately labile-Po														
6. Humic acid (Po)														
7. Fulvic acid (Po)	0.76**	0.91***		0.66*										
8. Soluble-P	0.80**	0.71**		0.68*			0.76**							
9. Ca_2_-P	0.91***	0.74**	0.72**	0.72**			0.70*	0.84***						
10. Ca_8_-P														
11. Al-P														
12. Fe–P	0.81**		0.73**	0.71**				0.67*	0.88***		0.61*			
13. Occluded-P							− 0.68*							
14. Ca_10_-P														
**Subsurface depth (0.2–0.4 m)**														
1. Labile-Pi														
2. Labile-Po	0.67*													
3. Microbial biomass P	0.60*													
4. Moderately labile-Pi														
5. Moderately labile-Po	0.70*	0.67*												
6. Humic acid (Po)														
7. Fulvic acid (Po)	0.84***	0.64*	0.65*	0.62*	0.65*									
8. Soluble-P	0.61*	0.81**		0.78**			0.69*							
9. Ca_2_-P	0.69*	0.74**	0.65*	0.59*			0.80**	0.67*						
10. Ca_8_-P														
11. Al-P	0.64*	0.60*					0.63*		0.81**					
12. Fe–P			0.64*						0.84***		0.76**			
13. Occluded-P			0.60*											
14. Ca_10_-P														

*Pi* inorganic P; *Po* organic P; Significance scale [* *p* < 0.05; ** *p* < 0.05; *** *p* < 0.05].

**Figure 4:**
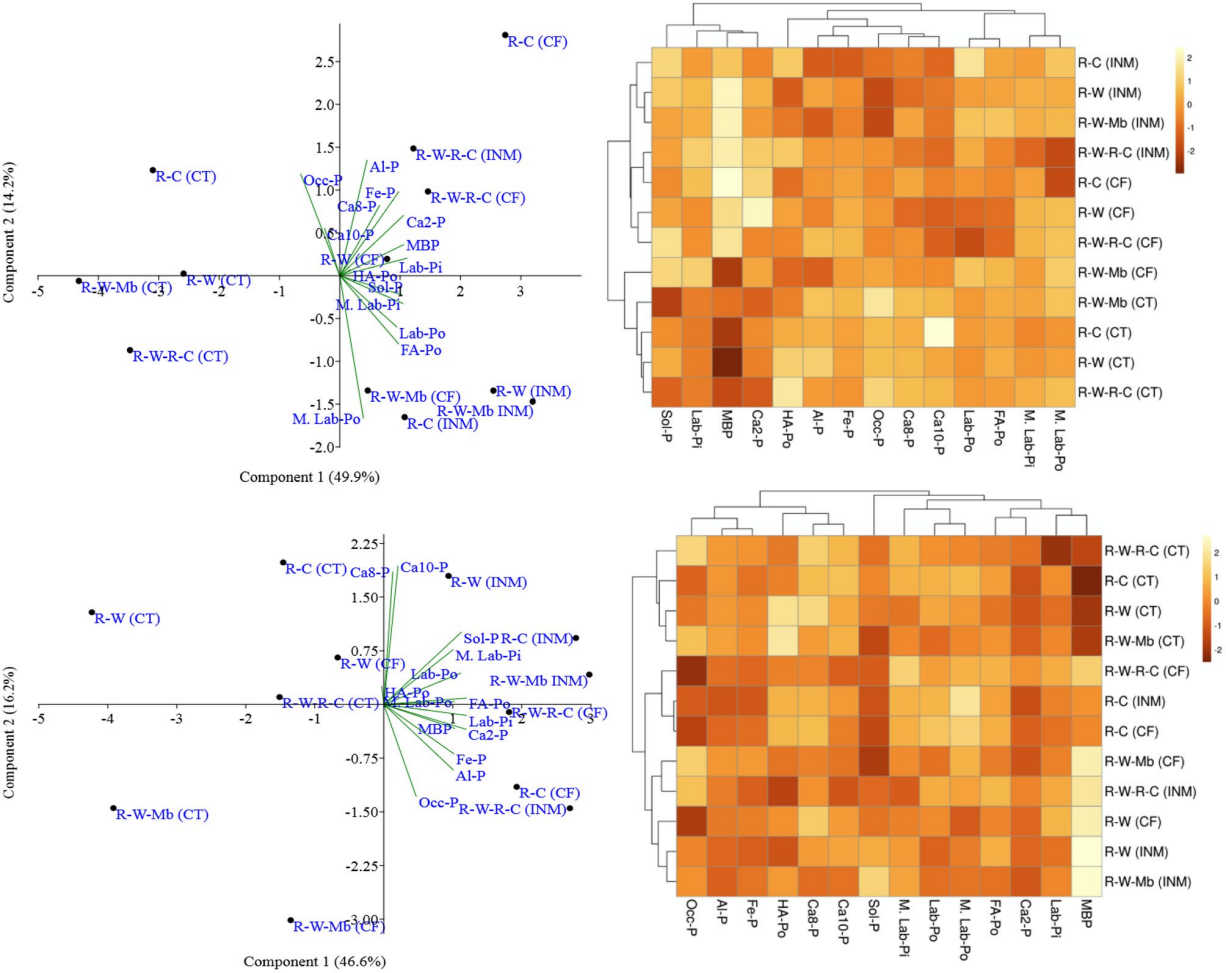
PCA scatter plot and heat-map cluster presentation of treatments based on soil P pools in surface (**a**) and subsurface (**b**) soil depths. *R–W*, rice–wheat; *R–W–Mb*, rice–wheat, mungbean; *R–W–R–C*, rice–wheat–rice–chickpea; *R–C*, rice–chickpea; *CT*, control; *INM*, integrated nutrient management; *CF*, sole chemical fertilizer treatment. *Sol-P*, soluble P; *Ca*_*2*_*-P*, di-calcium Phosphate; *Ca*_*8*_*-P*, octa-calcium phosphate; *Al-P*, Aluminium-bound P; *Fe-P*, Iron-bound P; *Occ-P*, occluded P;*Ca*_*10*_*-P*, octa-calcium P; *HA-Po*, humic acid Po fraction, *FA-Po*, fulvic acid Po fraction; *Lab-Pi*, Labile inorganic P pool; *Lab-Po*, Labile inorganic P pool; *MBP*, microbial biomass phosphorus; *M. Lab-Pi*,moderately labile inorganic P pool; *M. Lab-Po*, moderately labile organic P pool.

PCA results showed that the combination of R–C rotation with CF had the most significant impact (additive effect) on P fractions in the surface soil depth, while the control treatment of R–W–Mb had the lowest impact. The vector axis angle highlighted the close association of labile-P fractions with MBP, as well as a positive interrelation between labile-Po and moderately labile-P with occluded-P. In the subsurface soil depth, the combination of R–W–Mb with INM had the strongest positive impact on P pools, while R–W with CT had the lowest impact. Cluster plots clearly distinguished the effects of nutrient management treatments on P pools in both surface and subsurface soil depths (Fig. 4). Notably, there was a close association among control treatments for different rotations, forming tight clusters in both soil layers. Likewise, close associations among the sol-P, Ca_2_-P, labile-Pi and MBP were evident in both 0–0.2 m and 0.2–0.4 m depths. Multivariate regression analyses revealed that labile-Pi pool (sol-P + Ca_2_-P) was strongly associated with other inorganic P pools in both soil depths (Table 5). In surface soil depth, Fe-P, and in subsurface soil depth, Ca_8_-P and Al-P exhibited higher co-linearity with labile-Pi (*p* ≤ 0.05). The changes induced by treatments in labile organic and inorganic P significantly influenced the productivity of the base crop (rice), with the most substantial effect observed for labile-Pi (*p* ≤ 0.001) (Table 6). Furthermore, a strong positive association was observed between MBP and rice yield (*p* ≤ 0.001) in both soil depths.

**Table 5:** Multivariate regression equation explaining the linear relationship between dependent variable (labile-P) and independent variables (soil inorganic P pools).

Soil depth	Regression equation	Adjusted R^2^	*p* value	Soil P pools (X)	*SE*	*t*-stat	*p* value
0–0.20 m	Labile-P = 4.94–0.038Ca_8_-P–0.405Al-P + 2.74Fe-P–0.897Occluded-P + 0.022Ca_10_-P	0.66	0.033	Ca_8_-P	0.19	− 0.20	0.848
				Al-P	0.68	− 0.60	0.572
				Fe-P	0.85	3.21	0.018
				Occluded-P	0.51	− 1.75	0.131
				Ca_10_-P	0.16	0.15	0.889
0.2–0.4 m	Labile-P = − 39.1 + 2.9Ca_8_-P + 1.81Al-P + 0.92Fe-P + 0.33Occluded-P + 0.21Ca_10_-P	0.82	0.005	Ca_8_-P	8.51	3.17	0.019
				Al-P	0.09	2.52	0.045
				Fe-P	0.72	2.18	0.072
				Occluded-P	0.42	1.23	0.264
				Ca_10_-P	0.27	1.30	0.240

**Table 6:** Linear association (Y = a + bX) between soil P pools and base-crop (rice) productivity (Y) during the cropping year 2017–2018 (*n* = 36).

Soil depth	Soil P pools (X)	Correlation coefficient (*r*)	*p* value
0–0.2 m	Soluble-P	0.772	0.003
	Labile-Pi	0.935	< 0.001
	Moderately Labile Pi	0.852	< 0.001
	Labile-Pi + Moderately Labile Pi	0.929	< 0.001
	Labile-Po	0.767	0.004
	Moderately Labile Po	0.355	0.257
	Labile-Po + Moderately Labile Po	0.631	0.028
	Labile-Pi + Labile-Po	0.861	< 0.001
	Microbial biomass phosphorus	0.847	0.001
0.2–0.4 m	Soluble-P	0.418	0.177
	Labile-Pi	0.781	0.003
	Moderately Labile Pi	0.514	0.088
	Labile-Pi + Moderately Labile Pi	0.665	0.018
	Labile-Po	0.445	0.148
	Moderately Labile Po	0.582	0.047
	Labile-Po + Moderately Labile Po	0.580	0.048
	Labile-Pi + Labile-Po	0.563	0.056
	Microbial biomass phosphorus	0.873	< 0.001

## Discussion

### Inorganic P-pools

The Pi fractionation results reveal a prevalence of non-available forms, specifically Ca_8_-P and Ca_10_-P, while available P fractions, such as soluble-P and Ca_2_-P, are relatively low. This pattern is indeed characteristic of tropical alkaline soils that predominate in the IGP regions. The results underscore that the inclusion of chickpea in the R–W system could enhance soluble-P and Ca_2_-P levels, particularly in the subsurface soil depths (0.2–0.4 m)^40^. In all legume-inclusive rotations, the reduction in Ca_8_-P levels in the subsurface soil suggests that legumes possess a higher capacity to utilize less soluble forms of P, particularly higher in mungbean. Bafiec et al.^41^ have also reported that legumes contribute to a more substantial depletion of Ca_8_-P over time, depending on crop species and their P requirements. The strong equilibrium observed among soluble-P, Ca_2_-P, and Ca_8_-P in the soil system indicates that any inadequacy in labile Pi forms can lead to the chemical transformation of Ca_8_-P into labile Pi fractions (soluble-P and Ca_2_-P)^42^. Certainly, plant uptake of P is one of the principal factors for the evident changes in soil Pi pools. Considering the overall changes in soil Pi-fractions in control treatment, all the legume-inclusive rotations appear to have a more pronounced depleting effect compared to cereal–cereal rotation (R–W). This is likely attributable to the greater potential of legumes to tap into native P pools compared to cereals. As the control plots are completely devoid of nutrients, plant growth along with P removal by plants was lower in control plots (data not presented). Hence, it is conceivable that a true P omission plot could lead to a much higher degree of P depletion ^43^.

Our results demonstrated that INM enhanced bio-available Pi (both soluble-P and Ca_2_-P) when compared with CF in both the soil depths. Additionally, higher levels of Ca_10_-P are found in the control plot compared to INM and CF plots, indicating that plants are less efficient at utilizing this non-labile P form due to deficiencies in biochemical processes under nutrient deficiency conditions^44^. In the 0–0.2 m depth range, the reduction in occluded-P content under INM compared to control or CF plots suggests that the former either facilitates the dissolution of occluded-P or hinders P occlusion within sesquioxides^45^. The higher concentrations of Ca_2_-P, Ca_8_-P, and Fe-P in the CF-treated plots may be attributed to the gradual stabilization of these fractions into non-labile P forms due to the continuous application of P-fertilizers in CF plots, in contrast to the INM plots. Bhattacharya et al.^45^ reported that long-term INM practices strongly influence the sorption–desorption isotherm in rice soils, facilitating P desorption while reducing the extent of the sorption process. Therefore, the study suggests that replacing wheat with chickpea in R–W system could facilitate inorganic P mobilization in sub-surface soil depths, possibly attributed to increased dissolutions of Ca_8_-P. The results therefore highlighted the importance of INM to sustain a favorable soil inorganic P regime even with a suboptimal fertilizer P rate (half of the CF treatment).

### Lability-graded inorganic P pools

According to the results, substituting wheat with chickpea, either annually or on an alternating basis, leads to a notable enhancement of labile Pi pools, particularly within the subsurface soil layer. This improvement can be attributed to chickpea's inherent functional traits, including deep roots, soil acidification, and a higher release of carboxylates^10,46^. In contrast, mungbean, a fast growing legume (~ 60-day duration), may have efficiently utilized the native P pools, resulting in a significant reduction of labile Pi pools in the control treatment of R–W–Mb^47^. Moreover, the flooded rice ecologies offer an advantage by enhancing P bioavailability via increase in the solubility of occluded-P, Fe-P, Al-P, and Mn-P^48^. Consequently, rice plants can access various soil P fractions, potentially depleting the soil's P reserves over time if not adequately fertilized.

### Lability-graded organic P pools

In rotations involving chickpea (R–C, R–W–R–C), higher levels of labile-Po were observed compared to the R–W system, specifically in the subsurface soil. This increase in labile-Po was a result of higher soluble-P content, which facilitated the retention of labile-Po. While the content of fulvic acid-Po indicated that it is the predominant form of organic-P in the soil, it did not exhibit significant differences across various cropping systems^49^. The results clearly illustrate that INM enhances the bioavailability of P compared to sole chemical fertilizer treatments. This enhancement can be attributed to the substantial application of crop residue in the INM approach, facilitating P transformation and subsequent increases in various forms of labile P (soluble-P, labile-Pi, and moderately-labile-Po). The improved P availability in the INM approach may be attributed to significant biochemical changes in the soil^50^. These changes encompass the release of inorganic P from decomposing residues, the blockage of P sorption sites by organic molecules (specifically low molecular weight aliphatic acids) released from the residues, the regulation of soil pH, and the complexation of soluble Al and Fe by organic molecules. These mechanisms collectively contribute to the increased bioavailability of P in the INM treatment^50,51^. Moreover, it also plays a role in revitalizing the bioavailable P content in the soil. This includes increasing water-holding capacity, which promotes better root growth, enhancing micro-aggregation while reducing the number of potential P sorption sites, facilitating microbial immobilization of inorganic P, and causing short-term increases in soil pH. Consequently, these factors collectively contribute to an enhancement of bioavailable P content in the soil^52^.

The higher microbial biomass P along with higher available P in INM plots signifies an increase in microbial activity and the microbial assimilation of P^53^. Microorganisms, during the decomposition of residues, can release low molecular-weight organic acids. These acids compete with P for soil sorption sites, leading to an increase in soil solution P concentration^52,53^. Additionally, these organic acids may also reduce sorption sites through metal complexation and dissolution reactions, thereby releasing P for plant uptake^54^. The increased concentration of humic acid-Po in the surface soil depth and fulvic acid-Po in both surface and subsurface soil depths in INM plots may be attributed to the addition of organic matter. However, this addition also stimulates microbes to decompose the humic and fulvic acid fractions, thus releasing adsorbed P^55^. Results suggest that the rice ecology may have favored the stabilization of humic and fulvic acid fractions due to anaerobic soil conditions during the rice season^56^. Furthermore, the addition of crop residue in the INM approach provides physical protection to the soil by improving soil aggregation, resulting in the formation of stable clay-humate or fulvate complexes^57^.

The legume-inclusive crop rotations led to overall improvements in soil fertility, including KMnO_4_-N, NH_4_OAc-K, and SOC over the long run. In the 0–20 cm depth, pulse-based systems exhibited higher levels of available-N (8–29%), available-P (3–35%), available-K (6–15%), and available-S (3–13%) compared to the R–W system^57^. Results indicate that intensive cropping systems like R–W–Mb, without an adequate supply of P, may lead to a loss of fertility over time. However, a balanced application of organic residues alongside inorganic nutrients can not only enhance the bioavailability of P but also promote microbial activity.

### Surface–subsurface allocation of P pools

Typically, the fertility levels and microbial activity in the topsoil (0–0.2 m depth) are higher compared to those in subsurface soils, and this pattern extends to P content as well. However, the notably low SSBR values for soluble-P, Ca_2_-P, and moderately labile-Po in rotations involving chickpea (R–C and R–W–R–C) compared to the R–W rotation indicate that chickpea has a more positive impact on these pools in the subsurface soil as opposed to the surface soil^58^. This discrepancy may arise because of the dominant effects of the nutrient management treatments, potentially diluting the effects of crop rotation treatments^58^. Regarding nutrient management, the SSBR for occluded-P followed this pattern: CT > CF > INM (*p* ≤ 0.05). This suggests that INM may either impede the occlusion process or mobilize this pool into readily available forms for the plants, whereas P deficiency (control) might impair biochemical functions, resulting in greater occlusion within the sesquioxides^59^. In the CF plots, the prolonged application of synthetic P-fertilizers might have caused an increased SSBR for soluble-P, labile-Pi, and moderately labile Pi. The occluded P form, with the highest SSBR, implies that this pool is highly influenced by crop and soil management practices compared to the other fractions. Therefore, SSBR serves as a valuable index for assessing the relative dynamics of various Pi and Po fractions, and in the long-run and chickpea-based rotations with low SSBR values for labile-Pi (soluble-P and Ca_2_-P) indicate enrichment of subsurface P^58^.

### Correlations and multivariate analysis

**Figure 5:**
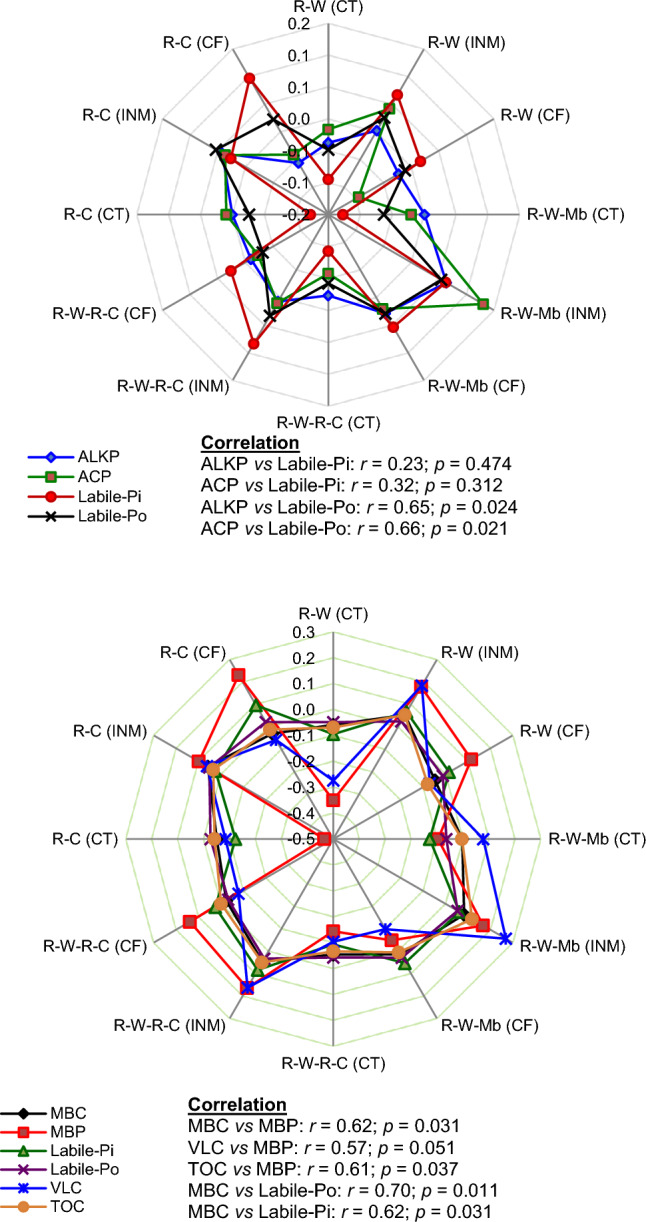
Z-score presentation to describe corresponding change in alkaline phosphatase (ALKP), acid phosphatase (ACP), microbial biomass carbon (MBC), and very-labile carbon (VLC) and their correlations with microbial biomass phosphorus (MBP), labile inorganic P (labile-Pi) and labile organic P (labile Po). *R–W*, rice–wheat; *R–W–Mb*, rice–wheat, mungbean; *R–W–R–C*, rice–wheat–rice–chickpea; *R–C*, rice–chickpea; *CT*, control; *INM*, integrated nutrient management; *CF*, sole chemical fertilizer treatment.

The negative correlation between occluded-P and bioavailable P pools (soluble-P, labile-Pi, and labile-Po) suggests the potential inter-conversion among these pools. According to the multivariate regression results, the relative concentrations of Fe-P in the surface soil and Ca_8_-P, Fe-P, and Al-P in the subsurface soil exert a significant influence on the labile-P pool. This indicates that increased P availability may be associated with the degree of P saturation in fixed forms. The results from PCA reveal that crop and nutrient management practices have differential impacts on bioavailable-P pools (soluble-P, microbial biomass phosphorus, labile-Pi, and labile Po) and fixed-P pools (Fe-P, Al-P, Ca_10_-P, and Ca_8_-P groups). Moreover, the distinctive nature of the occluded-P pool becomes apparent through PCA. In lowland rice soils subjected to flooding conditions, the dissolution of occluded-P is favored, and the application of organic matter might have further accelerated biochemical processes, leading to occluded-P depletion. Particularly in the subsurface soil, PCA coordinates clearly delineate the contrasting effects of different crop species and nutrient management practices. The PCA results also indicate that legume-inclusive rotations deplete overall P pools more than R–W rotations under P-deficient conditions (control treatment). This could be attributed to grain legumes exuding a higher amount of phosphatase enzymes compared to cereals^60,61^. Previous studies have reported that chickpea roots secrete larger quantities of acid phosphatase due to their increased demand for P compared to cereals^62^. Interestingly, our results suggest that phosphatase enzyme activity varies with the size of the labile-Po pool. However, the lack of a significant association between phosphatase enzymes and labile-Pi implies that bioavailable inorganic P does not necessarily regulate phosphatase enzyme activity (Fig. 5). This study observed an increase in phosphatase enzyme activity in the control treatments, consistent with previous reports indicating that a deficiency in plant-accessible Pi forms leads to increased phosphatase activity^63^. The differing responses of labile Pi and phosphatase enzyme activities to nutrient management treatments result in non-significant associations between these variable. Our results highlight that treatment induced improvement in soil labile P pools have significant positive influence on base crop (rice) productivity in long-run.

In the present context, the study findings offer promise for a sustainable and economically viable cropping approach for the farming community. According to the results, crop diversification, particularly when incorporating chickpea, emerges as a sustainable strategy to enhance soil fertility and overall soil health. This approach reduces the dependency on fertilizers, especially nitrogen (N), and minimizes water usage due to the lower water requirements of pulse crops. Moreover, it contributes to long-term improvements in the productivity of the base crop, resulting in direct benefits through reduced water and energy consumption. Conversely, an integrated approach to nutrient management holds the potential to enhance or maintain yields while reducing costs associated with fertilizer sources. It also serves to enhance soil functions and much relevant approach for the tropical alkaline soil conditions. Furthermore, this nutrient management approach presents a solution to the prevalent practice of burning cereal residues in the region, thereby mitigating environmental pollution.

## Conclusions

The study concluded that species diversification within rice-based systems can exert a notable influence on soil P bioavailability and the composition of P pools in lowland rice soils. Specifically, chickpea-inclusive rotations have demonstrated a favorable impact on P solubilisation with a more pronounced effect observed in the subsurface soil depth compared to the surface soil depth. Under conditions of P deficiency (P control), the R–W–Mb rotation significantly depleted the labile P pools, indicating an increased potential of the component crops to utilize native P pools. Furthermore, integrated nutrient management, which involves suboptimal nutrient doses, crop residue recycling, organic amendments, and bio-fertilizer application, has proven to enhance the mobilization of soil P over the sole chemical fertilization. This highlights the increased potential of crop residue recycling (or organic amendments) in enhancing P availability within alkaline rice soils. Notably, soluble-P, Ca_2_-P, and labile-P have established a strong equilibrium within the soil systems, while occluded-P, Fe–P, and Al-P exhibit a stronger co-occurrence within these pools. The study has unveiled a complex dynamic in the composition of different P pools, suggesting possible indirect influences of soil processes and functions on the relative composition of P pools within the soil system. Through the inclusion of legumes and the adoption of integrated nutrient management practices, soil P availability was improved, resulting in a substantial increase in base-crop productivity over time. In conclusion, the incorporation of legumes into rice-based production systems, particularly through chickpea-inclusive rotations, coupled with integrated nutrient management, emerges as a sustainable approach to enhance soil P availability and yield in the rice-based production systems of the northern Indo-Gangetic plains.

### Supplementary Information

Supplementary Figures.

## Data Availability

The datasets used and/or analyzed during the current study available from the corresponding author on reasonable request.
