# Supplementary material for: Long-term impact of legume-inclusive diversification and nutrient management practices on phosphorus dynamics in alkaline Fluvisol
Source: Sci Rep. 2024 Jan 2;14:65. doi: 10.1038/s41598-023-49616-x (PMC10762125; doi:10.1038/s41598-023-49616-x)
Supplement: Supplementary file 1 — Supplementary Figures. [file 41598_2023_49616_MOESM1_ESM.docx]

**Soluble-P Ca_2_-P Ca_8_-P Al-P Fe-P**

**Occluded-P Ca_10_-P Labile-Pi Labile-Po MBP**

**Moderately labile-Pi Moderately labile-Po Humic Acid Po Fulvic acid-Po**

**Supplementary Fig. 1** Interaction effect of crop rotation and nutrient management treatments on soil P pools in surface (0-0.2 m) soil depths. *a-g*, different lowercase letters are significant at *p* < 0.05 based on Duncan’s multiple range test. MBP, microbial biomass P.

**Soluble-P Ca_2_-P Ca_8_-P Al-P Fe-P**

**Occluded-P Ca_10_-P Labile-Pi Labile-Po MBP**

**Moderately labile-Pi Moderately labile-Po Humic Acid Po Fulvic acid-Po**

**Supplementary Fig. 2** Interaction effect of crop rotation and nutrient management treatments on soil P pools in subsurface (0.2-0.4 m) soil depths. *a-g*, different lowercase letters are significant at *p* < 0.05 based on Duncan’s multiple range test. MBP, microbial biomass P.
